# 2022 List of Reviewers

**DOI:** 10.1117/1.NPh.10.1.010102

**Published:** 2023-01-12

## Abstract

Thanks to reviewers who served Neurophotonics in 2022.

*Neurophotonics* would like to sincerely thank the following individuals who served as reviewers in 2022. The success of our publication hinges on the voluntary contributions of time and energy put forth by these professionals.

Androu Abdalmalak
Anna Letizia Allegra Mascaro
Juanita Anders
Jessica Anderson
Kamran Avanaki
Wesley Baker
Ramani Balu
Zeinab Barati
Adam Bauer
Borja Blanco
Pablo Blinder
Heather Bortfeld
Sabrina Brigadoi
Craig Brown
David Busch
Stefan Carp
Yi Chen
Jixin Cheng
Sangcheon Choi
Liam Collins-Jones
Ippeita Dan
Aniruddha Das
Ian Davison
Helga De Oliveira Miguel
Dominique Debanne
Michael Denton
Patrick Doran
Konstantin Doubrovinski
Patrick Drew
Adam Eggebrecht
Aykut Eken
Cameron Ellis
Joo Beom Eom
Sinem Erdogan
Baowei Fei
Tommaso Fellin
Claudio Ferre
Alissa Ferry
Ron Frostig
Xiaoxue Fu
Masahiro Fukuda
Yuanyuan Gao
Jessica Gemignani
Judit Gervain
Luca Giannoni
Sylvain Gigan
Ariel Gilad
Natalie Gilmore
Albert Gonzales
Grant Gordon
Gorm Greisen
Christine Grienberger
Dirk Grosenick
Yue Gu
Sabino Guglielmini
Lütfü Hanoğlu
Shi He
Brian Hill
Costantino Iadecola
Hongbo Jia
Yuanyuan Jiang
Jana Kainerstorfer
Kavon Karrobi
Isaac Kauvar
Hiroshi Kawaguchi
Pardis Kaynezhad
Anastasia Kerr-German
Kivilcim Kilic
Sohye Kim
Tiffany Ko
Michihiko Koeda
Sri-Rajasekhar Kothapalli
Baptiste Lacoste
Evelyn Lake
Frederic Lange
Nicole Lazar
Jerome Lecoq
Woeiming (Steve) Lee
Jon Levine
Sebastian Lewandowski
Rihui Li
Adam Liebert
Chao Liu
Ning Liu
Antoine Louveau
Anna Martinez
Giovani Martins
Paul Mazaika
Rodrigo Menezes Forti
Rickson Mesquita
Daniel Milej
Anusha Mishra
Takeyuki Miyawaki
Samuel Montero-Hernandez
Satoshi Morimoto
Jutta Mueller
Timothy Murphy
Simon Musall
Andrew Nelson
Thien Nguyen
Haijing Niu
Jack Noah
Sergio Novi Junior
Hellmuth Obrig
Ian Oldenburg
Wiktor Olszowy
Yumie Ono
Felipe Orihuela-Espina
Antonio Ortega-Martinez
Leif Ostergaard
Ashwin Parthasarathy
Gulcin Pekkurnaz
Ke Peng
Stephane Perrey
Paola Pinti
Luca Pollonini
Lesley Probert
Vidhya Rangaraju
Rebecca Re
Mitchell Robinson
Sara Sanchez-Alonso
Mark Sanders
Hendrik Santosa
Angelo Sassaroli
Hiroki Sato
Christopher Schaffer
Felix Scholkmann
Andy Shih
Mohinish Shukla
Justin Skowno
Spencer Smith
Keith St. Lawrence
Stefan Stamenkovic
Bojana Stefanovic
Ulas Sunar
Susanna Tagliabue
Jianbo Tang
Tong Boon Tang
Fenghua Tian
Nicholas Todd
Yunjie Tong
Alessandro Torricelli
Daisuke Tsuzuki
Ernesto Vidal Rosas
Alexander von Luehmann
Hui Wang
Martin Wolf
Jiamin Wu
Jingyi Wu
Melissa Wu
Yicong Wu
Lei Xi
Yi Xue
Jinyao Yan
Hongki Yoo
Mark Zarella
Fen Zhang
Xian Zhang
Zihui Zhang
Jiangbing Zhou
Xiaoqing Zhou
Hamoon Zohdi

